# Previous BCG vaccination is associated with less severe clinical progression of COVID-19

**DOI:** 10.1186/s12916-023-02859-x

**Published:** 2023-04-13

**Authors:** Susan Martins Pereira, Florisneide Rodrigues Barreto, Ramon Andrade de Souza, Carlos Antonio de Souza Teles Santos, Marcos Pereira, Enny Santos da Paixão, Carla Cristina Oliveira de Jesus Lima, Marcio Santos da Natividade, Ana Angélica Bulcão Portela Lindoso, Eder Gatti Fernandes, Evonio Barros Campelo Junior, Julia Moreira Pescarini, Kaio Vinicius Freitas de Andrade, Fernanda Mattos de Souza, Elisangela Alves de Britto, Ceuci Nunes, Maria Yuri Ichihara, Margareth Dalcolmo, Anete Trajman, Manoel Barral-Netto, Ibrahim Abubakar, Mauricio Lima Barreto, Ricardo Arraes de Alencar Ximenes, Laura Cunha Rodrigues

**Affiliations:** 1 Institute of Collective Health, Federal University of Bahia, Salvador, Brazil; 2 Center for Data and Knowledge Integration for Health (CIDACS), Fiocruz, Salvador Brazil; 3 Department of Exact Sciences, State University of Feira de Santana, Bahia, Salvador Brazil; 4 Faculty of Epidemiology, London School of Hygiene and Tropical Medicine, London, UK; 5 Institute of Infectology, Emílio Ribas-SES-SP, São Paulo, Brazil; 6 Department of Tropical Medicine, Health Sciences Center, Federal University of Pernambuco, Recife, Brazil; 7 Health Department, Universidade Estadual de Feira de Santana, Feira de Santana Bahia, Brasil; 8 Institute of Social Medicine, University of the State of Rio de Janeiro, Rio de Janeiro, Brazil; 9 State Department of Health of Bahia, Institute Couto Maia, Salvador, Brazil; 10 Hélio Fraga Reference Center, Fundação Oswaldo Cruz (Fiocruz), Rio de Janeiro, Brazil; 11 Departamento de Clínica Médica, Universidade Federal do Rio de Janeiro, Rio de Janeiro, Brazil; 12 McGill TB International Centre, McGill University, Montreal, Canada; 13 Institute Gonçalo Moniz - Fundação Oswaldo Cruz (Fiocruz), Salvador, Brazil; 14 Faculdade de Medicina, Federal University of Bahia, Salvador, Brazil; 15 Institute for Global Health, University College London, London, UK

**Keywords:** BCG, Bacillus Calmette-Guérin, COVID-19, SARS-CoV-2, Coronavirus disease 2019, Severity, Vaccination

## Abstract

**Background:**

BCG vaccination, originally used to prevent tuberculosis, is known to “train” the immune system to improve defence against viral respiratory infections. We investigated whether a previous BCG vaccination is associated with less severe clinical progression of COVID-19

**Methods:**

A case-control study comparing the proportion with a BCG vaccine scar (indicating previous vaccination) in cases and controls presenting with COVID-19 to health units in Brazil. Cases were subjects with severe COVID-19 (O2 saturation < 90%, severe respiratory effort, severe pneumonia, severe acute respiratory syndrome, sepsis, and septic shock). Controls had COVID-19 not meeting the definition of “severe” above. Unconditional regression was used to estimate vaccine protection against clinical progression to severe disease, with strict control for age, comorbidity, sex, educational level, race/colour, and municipality. Internal matching and conditional regression were used for sensitivity analysis.

**Results:**

BCG was associated with high protection against COVID-19 clinical progression, over 87% (95% CI 74–93%) in subjects aged 60 or less and 35% (95% CI − 44–71%) in older subjects.

**Conclusions:**

This protection may be relevant for public health in settings where COVID-19 vaccine coverage is still low and may have implications for research to identify vaccine candidates for COVID-19 that are broadly protective against mortality from future variants. Further research into the immunomodulatory effects of BCG may inform COVID-19 therapeutic research.

## Background

Bacillus Calmette-Guérin (BCG) vaccine is safe and low-cost. Currently, recommendations for BCG vaccination are based on local incidence of tuberculosis (TB) at birth, in childhood, or for target groups.

BCG vaccination has well-known non-specific effects. These can be therapeutic or preventive. Immunotherapy with BCG has been the standard treatment for bladder cancer for 50 years [1]. Similarly, BCG vaccination can protect against early mortality in low-income/high-infant mortality countries, probably via protection against respiratory viral infections [2–5], including respiratory syncytial virus [6]. It is suggested that the non-specific effect of BCG is mediated through immunomodulation or “trained immunity” [4, 7, 8].

Given the evidence for non-specific effects of BCG against viral respiratory infections, the hypothesis that BCG vaccination might offer some protection against COVID-19 was raised early in the epidemic, and a number of analyses of existing data were conducted, with conflicting results [9–13]. More recently, original research was conducted, again with conflicting results [14–22].

The reason for conflicting results is not clear. It is of interest that in studies of COVID-19-specific vaccines, protection against infection, clinical progression, or severe or fatal forms differ. Likewise, the protection conferred by BCG against tuberculosis is complex and differs according to whether it is protection against infection or progression from infection to disease [23] against different forms and whether it is given at birth/to people never exposed or at a later age/after exposure to non TB mycobacteria [24, 25].

The biological mechanism behind BCG reduction in non-specific morbidity and mortality, including against COVID-19, is poorly understood, with ongoing experimental research and theoretical explanations put forward [16, 18, 26–28].

Here, we present the results of an epidemiological study estimating the effect of neonatal BCG vaccination on reducing the risk of clinical progression from symptomatic COVID-19 to severe forms of the disease in individuals who did not receive a COVID-19 vaccination.

## Methods

### Study design

Initially, the study design is unmatched case-control study. We conducted an unplanned sensitivity analysis, with subsequent strict internal matching. Data collection was carried out during 2020 and 2021.

### Study population

The study population is cases of COVID-19 (confirmed by positive RT-PCR) presenting at COVID-19 referral hospital/health care units in the cities of Salvador, BA; São Paulo, SP; and Recife, PE, with no age restriction.

### Exclusion criteria

Past vaccination against COVID-19. Recruitment started before vaccination against COVID-19 was available; after vaccination was introduced, subjects who had received any dose of any COVID-19 vaccine were not recruited. COVID-19 vaccination status was ascertained by card or patient history.

### Case definition and recruitment

Individuals were hospitalised in intensive care units with severe COVID-19. Severity was defined according to the Brazilian Ministry of Health clinical management protocol for hospitalisation for COVID-19: O_2_ saturation < 90%, severe respiratory effort, severe pneumonia, severe acute respiratory syndrome (SRAG), sepsis, and septic shock [29].

### Control definition and recruitment

Individuals with COVID-19 present at the same health unit as cases who did not meet the above criteria for severity and were not hospitalised or were hospitalised in general wards. An exception was the final period of data collection in the city of Salvador, Bahia. The referral hospital in Salvador modified its routine to receive only severe COVID-19 patients—i.e. only those who met the criteria to be a case in the study. During this period in Salvador, controls were recruited at a primary attention referral centre that was receiving subjects with COVID-19 that did not require intensive care. A sensitivity analysis was conducted excluding cases and controls from Salvador.

### Exposure variable

Past intradermal BCG vaccination status was ascertained through the examination of the left or right upper arm for the presence or absence of a BCG vaccine scar.

### Post hoc internal matching for sensitivity analyses

City, age (within 2 years interval or less), and presence of any comorbidity.

### Data collection

Examination of the arm for a BCG scar was conducted on admission to the health unit, by a doctor or nurse from the staff. In case of doubt, the research team member responsible for the local data collection carried out another inspection. The ascertainment of BCG scar was conducted before the patients were classified as cases or controls. We did not enquire about the history of oral BCG. Information on the date of onset of symptoms and clinical, epidemiological, and laboratory data was collected from medical records, in consultation with the laboratory environment manager and when necessary, from telephone contacts.

### Sample size

The required sample was estimated as 1250 (625 cases and 625 controls) using Kelsey’s formula for unpaired case-control studies [30] and considering an alpha value of 5%, power 80%, exposure (BCG scar) in controls 65%, vaccine efficacy 30%, and the same number of cases and controls, with 20% losses.

### Analyses

The summary OR was estimated using the Mantel-Haenszel method for unpaired samples. Modelling with logistic regression used unconditional regression analysis to control for age (in 10-year intervals), race/colour, sex, educational level, presence of comorbidity, and city; sensitivity analysis was conducted using conditional regression analysis after rigorous matching of cases and controls by age (2 years of age difference or less), presence or absence of comorbidity, and municipality. Interactive terms were added to investigate if protection varied with age and with the presence of comorbidity. The protective vaccine efficacy (VE) was 1 − OR. To fit the best model, Akaike estimator was performed [31].

## Results

We included 497 cases and 670 controls, from three Brazilian cities (São Paulo, SP; Salvador, BA; Recife, PE). The mean age for cases and controls were 55.8 [± 18.3] and 41.3 [± 14.7], respectively. Cases were older than controls (41.8% of cases and 9.3% of controls were aged above 60); 59.6% of the cases and 43.2% of the controls were male. 58.7% of cases and 92.1% of controls had a BCG scar. Controls had higher education levels (33.0%) than cases (11.8%). Brown race/colour was predominant in both cases (70.1%) and controls (47.6%). Comorbidity was present in 80.2% of cases and 33.8% of controls. For all variables above, the differences were statistically significant (Table 1). The crude odds ratio (OR) of BCG vaccination was 0.12 (95% confidence interval (95% CI): 0.09–0.17) corresponding to an overall protective effect of 88% (95% CI: 83.0–91.3%). After adjusting by race/colour, schooling, sex, age, municipality, and presence of comorbidity, the OR was 0.27 (95% CI: 0.17–0.44) corresponding to an overall protective effect of 73% (95% CI: 56.0–83.0%) (Table 2). In a sensitivity analysis using matched data on age and comorbidity, the overall crude protective effect remained similar, 65%, OR= 0.35 (95% CI: 0.21–0.58) and 72% OR = 0.28 (95% CI: 0.13–0.58), when further adjusted for sex, education, and race/colour. In an analysis of matched data on age, comorbidity, and municipality, the overall crude protective effect was 70%, OR = 0.30 (95% CI: 0.17–0.53). After adjusting by race/colour, schooling, and sex, this protective effect was 70% OR = 0.30 (95% CI: 0.14–0.63) (Table 2).

**Table 1:** Distribution of COVID-19 cases and controls, in different cities in Brazil, 2020–2021

**Variable**	**Cases (*N* = 497)**		**Controls (*N* = 670)**		***P*-value**
	***n***	**%**	***n***	**%**	
**Age**					< 0.000
0–30	45	9.0	180	27.0	
31–40	43	8.6	172	25.8	
41–50	85	17.1	128	19.2	
51–60	117	23.5	124	18.6	
61 +	208	**41.8**	62	**9.3**	
**Sex**					< 0.000
Female	201	40.4	376	56.8	
Male	276	59.6	286	43.2	
**BCG scar**					< 0.000
Yes	292	58.7	617	92.1	
No	205	41.2	53	7.9	
**Education**					< 0.000
Never	39	8.9	9	2.0	
Elementary school	207	47.0	97	21.8	
High school	142	32.3	192	43.1	
Higher education	52	11.8	147	33.0	
**Race/colour**					< 0.000
White	113	22.8	251	38.1	
Black	24	4.8	85	12.9	
Brown	347	70.1	314	47.6	
Yellow	11	2.2	9	1.4	
**Municípality**					< 0.000
Salvador and Metropolitan region, BA	237	47.3	284	42.3	
Recife, PE	120	23.9	170	25.3	
São Paulo, SP	144	28.7	217	32.3	
**Comorbidity**					< 0.000
Yes	390	80.2	177	33.8	
No	95	19.5	295	56.4	
**Missing**	**1**	0.2	51	9.7

**Table 2:** Association between COVID-19 and covariate-adjusted past BCG vaccination using logistic regression

**Unmatched**^**a**^** (*N* = 1167)**		**Matched**^**b**^** (200 pairs matched by age and presence of comorbidity) (*N* = 400)**		**Matched**^**b**^** (203 pairs matched by age, presence of comorbidity, and city) (*N* = 406)**	
OR (95% CI)		OR (95% CI)		OR (95% CI)	
Crude	Adjusted	Crude	Adjusted	Crude	Adjusted
0.12 (0.09–0.17)	0.27 (0.17–0.44)	0.35 (0.21–0.58)	0.28 (0.13–0.58)	0.30 (0.17–0.53)	0.30 (0.14–0.63)

^a^Adjusted by race/colour, schooling, sex, age, municipality, and comorbidity

^b^Adjusted by race/colour, schooling, and sex

The protective effect of BCG vaccination was higher in those without comorbidities 85% (95% CI: 71.0– 91.6%) than in those with comorbidities 76% (95% CI: 62.0–85.0%). Table 3 shows that the protective effect was low in those aged over 60, 35.0% (95% CI: − 44–71%), and very high in those aged 60 years or less, 87% (95% CI: 74.1–93.1%) (Table 3).

**Table 3:** Efficacy of BCG against severe COVID-19 by age group, Brazil, 2020–2021

**Age group**	**0–60**	**0–30**	**31–40**	**41–50**	**51–60**	**61+**
**Eficacy (95% CI)**^a^	87.0% (74.1–93.1%)	97.1% (25.4–99.9%)	90.0% (21.2–98.7%)	84.0% (42.6–95.3%)	81.0% (48.2–92.7%)	35.0% (− 44.4–70.7%)

^a^Adjusted by race/colour, schooling, sex, municipality, and comorbidity

## Discussion

In our study, a previous intradermal BCG vaccination indicated by the presence of scar conferred protection against progression to severe COVID-19 in (COVID-19 unvaccinated) subjects in all age groups, 87% in individuals aged 60 or less, and 35% in older subjects.

The study has some strengths and limitations. Case-control studies are vulnerable to bias, which in this study would have been introduced if ascertainment of BCG vaccination was different in cases and controls. This was prevented by ascertaining the presence of BCG vaccination scar at arrival at the referral hospital/health unit for individuals with COVID-19, before they were allocated to an ICU (more severe, cases) or not (controls). An exception was the final period of data collection in the city of Salvador. Because of a change in admission policy, the final controls were selected from a referral centre who was receiving subjects with COVID-19 that did not require intensive care; sensitivity analysis including and excluding those showed very little change.

Another related concern is whether BCG scar is a good indicator of previous intradermal BCG vaccination. There is good evidence that scar correlates well with intradermal vaccination until at least the age of 14 years in Brazil [32]. Even if scars were not a good indicator of previous intradérmic BCG vaccination, and this was unrelated to the subject being a case or a control, such nondifferential misclassification could only underestimate vaccine protection in the control group [33].

Another potential limitation is that we used scar as the only indication of previous BCG vaccination; information on oral BCG was not collected. Intradermal BCG was introduced in Brazil in 1968; so individuals born before that year, who would have been 56 or over at the time of the study, could have received oral BCG. This has two implications: first, the few people aged 56 or older in the study classified as vaccinated had a BCG scar, so they must have received intradermal BCG, either at school age, during a catch-up vaccination, or because a potential exposure, for example, as a worker in health care settings. Second, if oral BCG (like intradérmic BCG) has an effect in decreasing COVID-19 severity, and a proportion of people aged 56 or older without a BCG scar had received oral BCG, this would artificially decrease the estimated effect of BCG scar in the older group.

A second potential vulnerability in case-control studies is selection bias, which is introduced when the selection of cases is influenced by exposure status. In this study, this would have been introduced if knowledge of BCG vaccination influenced whether a subject was selected as a case or a control. Classification into case or control followed a rigorous objective criteria based on measurable clinical and laboratory data as cases and controls were enrolled at the hospital emergency room/admission, so selection bias is unlikely. Data related to COVID-19 infection was retrieved from medical records available at the hospitals; they were mostly completed independent of enrollment as a case or not in the study. Differences in characteristics associated with COVID-19 severity—age, presence of comorbidity—were very marked between cases and controls as expected from clinical data. This was addressed initially by controlling in the analyses; the efficacy remained high in a sensitivity analysis conducted matching internally closely for age, comorbidity, and city. Residual confounding is not a likely explanation to our findings as the protective effect remained remarkably stable in the sensitivity analysis with cases and controls closely matched by age, co-morbidity, and municipality. Finally, our study did not measure BCG immunological correlates of protection of BCG as none is known.

How to interpret the strong variation in protection with age? We offer four possible explanations. First, the small number of subjects in the older age group. Since intradermal BCG vaccination was introduced in Brazil, the proportion of people over 60 years vaccinated with BCG is small, and thus, the power of the study to detect protection in this age group was limited. Second, the possibility that individuals in that age group considered unvaccinated because they did not have a BCG scar had in fact received oral BCG; this would artificially decrease the measured protection. Third, there is evidence that the duration of immunity conferred by BCG decreases with time [24, 34]. Finally, the severity of COVID-19 increases very markedly in those aged 60 and over, and the effect BCG on preventing progression may interact with the biological mechanism behind the effect of age on COVID-19 severity.

Although a number of studies of BCG preventing clinical progression of symptomatic COVID-19 to severe COVID-19 were conducted, with varying results, this is by far the largest conducted with original data. Our results are broadly consistent with two other large studies examining the effect of previous BCG on clinical progression. The first, conducted by Weng, followed a cohort of 120 COVID-19 patients, predominantly of Hispanic origin, in the USA. The study was completed before COVID-19 vaccine was introduced, so study participants were unvaccinated [18]. Eighty-two participants (68.3%) had a previous BCG vaccination. Individuals with BCG vaccination were less likely to require hospital admission during the disease course (3.7% vs. 15.8%, *P* = 0.019), after adjusting for demographics and comorbidities. This corresponds to a protection of 76% against clinical progression to severity (our calculation). The second, conducted by Sinha [35] is a multi-centre quadruple-blind, parallel assignment randomised control trial. It was found as a secondary outcome that significantly more patients in the placebo group (who did not receive BCG) progressed to severe COVID-19 pneumonia and required hospitalisation and oxygen [35]. These results suggest that BCG protection against clinical progression is so similar in these 3 studies, given that two estimated the effect of previous BCG and the third had BCG as a recent intervention.

While the mechanism of BCG vaccine-related reductions in non-specific morbidity and mortality is poorly understood, there are several postulated explanations. First is trained immunity, where BCG vaccination induces epigenetic reprogramming of monocytes through histone modifications in regulatory elements of specific genes, resulting in increased cytokine production following subsequent exposure to pathogens [36]. Second, in addition to this innate immune mechanism, an adaptive immune response through cross-reactivity and bystander activation of T cells may have a role. Cross-reactivity involves a reaction to a different antigen with amino acid similarity (BCG contains similar 9-amino acid sequences with SARS-CoV-2) [37], while a bystander response occurs via a neighbouring, unrelated T cell with a different specificity to the one using involved. Although the exact duration of protection based on either mechanism is not known, strong production of Th1 and Th17 immune response to non-mycobacterial antigens in trained immunity [38] and a specific BCG-associated DNA methylation signature involved in viral response pathways have been reported for over a year after BCG vaccination. Cellular immunity is an important determinant of COVID-19 disease outcomes and infections caused by coronavirus infections more broadly [39]. Indeed, multiple COVID-19 vaccines and prior infection by SARS-CoV-2 variants provide protection against severe outcomes following subsequent exposure. There is evidence of virus-specific cellular responses without virus-specific antibodies suggesting that in some individuals [40, 41], an infection may be cleared by the cellular immune system before it is fully established. Consequently, it is plausible that the non-specific effects of BCG vaccination may also have a role in initial protection and the subsequent clinical course of COVID-19.

## Conclusions

In conclusion, our results suggest a protective effect of the BCG vaccine against progression to severe COVID-19 in people who did not receive COVID-19 vaccines. This confirms the findings of the two other large studies. More studies to be conducted in different epidemiological scenarios are needed to confirm or rule out this specific strong association. In addition to the protective effect against clinical progression, relevant research questions should explore whether the time elapsed since BCG vaccination and age at BCG vaccination affect protection and whether this explains the lack of protection in older people in our study. It would also be interesting to investigate if the same protection is present in people who received COVID-19 vaccines. We reiterate the importance of discriminating studies that estimate the protection of BCG vaccination against infection with COVID-19, symptomatic COVID-19, clinical progression to severity, and severe disease. We hope that the specificity of the finding (protection against progression) might inform the immunological study of non-specific effects of BCG and the mechanisms behind the marked increase in the severity of COVID-19 in the elderly.

In our view, it is too soon to recommend BCG vaccination to help prevent severe COVID-19, but if protection is confirmed, it may be an alternative, particularly in settings with low COVID-19 vaccine coverage. Our findings may also have implications for research to identify vaccine candidates for COVID-19 that are broadly protective against mortality from future variants. Future work to understand the immunomodulatory effects of BCG may also inform therapeutic research.

## Data Availability

The datasets used and/or analysed during the current study are available from the corresponding author (florisneide@gmail.com) upon reasonable request.

## Abbreviations

BCG: Bacillus Calmette-Guérin
CI: Confidence interval
O2: Oxygen
OR: Odds ratio
RT-PCR: Reverse transcriptase polymerase chain reaction
SRAG: Severe acute respiratory syndrome
